# Dislocation-Governed Plastic Deformation and Fracture Toughness of Nanotwinned Magnesium

**DOI:** 10.3390/ma8085250

**Published:** 2015-08-13

**Authors:** Lei Zhou, Ya-Fang Guo

**Affiliations:** Institute of Engineering Mechanics, Beijing Jiaotong University, Beijing 100044, China; E-Mail: 09115277@bjtu.edu.cn

**Keywords:** nanotwinned magnesium, molecular dynamics, twin boundaries, plastic deformation, fracture toughness

## Abstract

In this work, the plastic deformation mechanisms responsible for mechanical properties and fracture toughness in $\{10\bar{1}2\}<10\bar{1}\bar{1}>$nanotwinned (NT). magnesium is studied by molecular dynamics (MD) simulation. The influence of twin boundary (TBs) spacing and crack position on deformation behaviors are investigated. The microstructure evolution at the crack tip are not exactly the same for the left edge crack (LEC) and the right edge crack (REC) models according to calculations of the energy release rate for dislocation nucleation at the crack tip. The LEC growth initiates in a ductile pattern and then turns into a brittle cleavage. In the REC model, the atomic decohesion occurs at the crack tip to create a new free surface which directly induces a brittle cleavage. A ductile to brittle transition is observed which mainly depends on the competition between dislocation motion and crack growth. This competition mechanism is found to be correlated with the TB spacing. The critical values are 10 nm and 13.5 nm for this transition in LEC and REC models, respectively. Essentially, the dislocation densities affected by the TB spacing play a crucial role in the ductile to brittle transition.

## 1. Introduction

Strength and ductility are two important mechanical properties pursued by industry applications. Currently, nanocrystalline (NC) metals and alloys are widely studied for their superb mechanical properties with grain refinement into the nano-scale, which is mostly less than 100 nm. Because of the introduction of interface defects such as grain boundaries (GBs), coherent twin boundaries (CTBs) and incoherent twin boundaries (ICTBs) into macrostructure, strength is significantly enhanced since these defects serve as dislocation sources which provide sufficient dislocation nucleation sites along the interfaces [1,2,3,4]. However, dislocation mobility is also impeded by GBs. As a result, the localized stress can concentrate on the GBs and then make it favor the crack propagation [5]. In contrast to the GBs, CTB has a lower interface energy and is more vulnerable for the nucleation and motion of dislocations. For the purpose of improving the combination of strength and ductility, nanotwinned (NT) materials consisting of a lamella structure, namely grains with twin boundaries (TBs) parallel to each other underlying the microstructure, are synthesized. To date, NT Cu has been widely synthesized [6,7]. However, magnesium, a newly used metal for its unique properties such as low density and relatively high strength, is rarely mentioned for use in NT materials. Existing experiments showed that NT structure in magnesium alloys could be obtained by applying a tensile loading or using surface mechanical attrition treatment (SMAT) [8,9]. Recently, Pozuelo *et al.* [10] proposed a comprehensive approach to form NT structure in NC Mg-based alloys.

In NT Cu, it has been reported that there are different TB densities, as TB spacing results in a transition in the yielding mechanism from the interaction between TBs and dislocations to multiple dislocation activities [11]. With the refinement of grains, the interaction between TBs and dislocations gradually dominates the strain hardening. Thus, the capacity of dislocation storage is highly enhanced by the strength of NT Cu reaching its maximum at a critical value of TB spacing, about 15 nm. In NT magnesium, mechanical properties of magnesium alloy can be improved via pre-twinning [12]. Recently, using the molecular dynamics (MD) method, Song *et al.* [13] investigated the temperature and TB spacing effects on strength of NT magnesium. It has been reported that the basal dislocation emissions dominate the plastic deformation. Moreover, dislocation storage ability was also pronounced a key role to the yield strength shift through changing TB spacing as well as the repulsive force between TBs and dislocations.

In addition to promoting yielding strength, more and more attention is currently focused on the fracture toughness of NT materials. It is well known that avoiding the crack initiation or providing high resistance to the crack propagation will improve the fracture toughness. Experiment results [14] showed that parallel TBs protect dimples from coalescing which serve as crack initiation sites in bulk nanostructure Cu. Also, because of the dislocation emission from interface defects, crack is likely to be blunted or deflected if they propagate towards TBs. Therefore, the NT structure improves the local plasticity in bulk Cu. However, the existent research on fracture toughness of NT materials mainly focused on FCC Cu. Because of the limited numbers of slip systems in hexagonal close-packed (HCP) materials, the deformation mechanism in NT Mg seems not similar to those in FCC metals. According to previous experiments [15,16], dislocation slip played a significant role in plastic deformation of pre-twinning magnesium alloy. moreover, basal dislocation motions can also occur at the crack tip, thereby affecting the crack growth [17] and they also potentially being arrested by the TBs [18]. it is thus necessary to reveal the effects of dislocation motions on the crack propagation in nanotwinned magnesium.

In the present work, we create models with the pre-existing crack to investigate mechanical properties and plastic deformation mechanisms of NT magnesium. Crack positions and TB spacing variations are addressed. We aim to get a better insight of the fracture toughness by studying the factors affecting the crack propagation.

## 2. Simulation Models and Method

**Figure 1:**
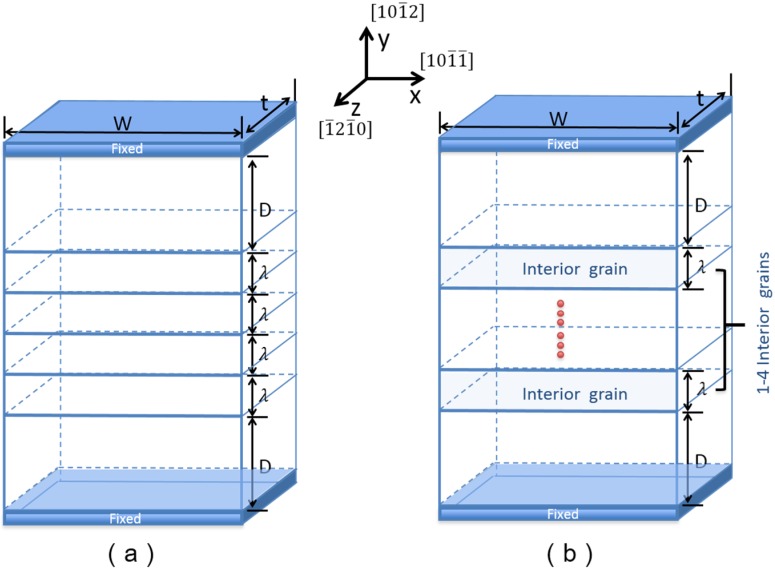
A schematic diagram of two types of MD models: (**a**) type A; (**b**) type B.

As shown in Figure 1, two types of models are adopted to perform MD simulations at the nano-scale. In type A, five TBs are contained in the model, while TB spacing (λ) varies in the simulation. In type B, TB spacing (λ) keeps a constant (7.5 nm), while different numbers of grains (1–4) are contained. Moreover, the sizes of the top and bottom grains on the models are larger than that of the interior grainin order to reduce the surface effect. We use the $\{10\bar{1}2\}<10\bar{1}\bar{1}>$ > TB in our simulations, which is the most common TB in magnesium [19,20]. An elementary unit of NT magnesium is set up by rotating two single magnesium crystal grains according to the $\{10\bar{1}2\}<10\bar{1}\bar{1}>$ > twinning orientational relationship. Then, we duplicated the elementary unit several times to get an NT model which contained several $\{10\bar{1}2\}<10\bar{1}\bar{1}>$ > TBs within it. The *x*-, *y*- and *z*- direction lies along $\left[10\bar{1}\bar{1}\right]$ >, $\left[10\bar{1}2\right]$ and $\left[\bar{1}2\bar{1}0\right]$ , respectively, as shown in Figure 1. Free boundary conditions are applied to *x*- and *y*-direction; periodic boundary condition is assigned to the *z*-direction for simulating the mode I crack.

Table 1 presents parameters of type A models. According to the experimental results reported by Yu *et al.* [9], we introduce six different dimensions of the interior grains in y-direction ranging from 5 to 15.5 nm to investigate the TB spacing effect on the plastic deformation mechanisms in type A. Considering the effect of crack position in samples, two types of crack were employed in type A—the left edge crack (LEC) and right edge crack (REC)—by removing several atom layers along the TBs. The intergranular crack length is all about 4 nm. In type B models, the number of interior grains is changed from 1 to 4, while TB spacing is a constant 7.5 nm. Only LEC is studied in type B.

**Table 1:** Parameters of type A models.

λ (nm)	D (nm)	W (nm)	t (nm)	Number of Atoms
5	16	40	2.4	159,368
7.5	16	40	2.4	191,368
10	16	40	2.4	235,520
12	16	40	2.4	268,088
13.5	16	40	2.4	299,720
15.5	16	40	2.4	320,256

These two models were simulated in constant NPT ensemble with a velocity-Verlet integrator. The temperature was kept at 5 k and the pressure in z-direction was controlled at 0 bar by the Nosé–Hoover thermostat. We employed the embedded atom method (EAM) developed for magnesium by Liu *et al.* [21] to describe the atomic interactions. This potential has been proved useful in investigating the dislocation nucleation and twinning behavior by many studies [22,23,24]. The molecular dynamics program LAMMPS [25] was employed in simulations. The common neighbor analysis (CNA) method [26] and Atomeye software [27] were used for visualizing the evolution of the atomistic structures.

After introducing an intergranular crack in the model, we first relaxed the initial simulation system (both type A and type B) 10,000 steps to achieve the equilibrium state. Then, uniaxial tensile loadings were applied by imposing tensile displacements to atoms in the fixed layers at the top and the bottom of models along the y-direction. The systems were relaxed for 5000 steps after every 0.3% stretch strain applied. A constant strain rate 1 × 10^8^ s^−1^ was applied to simulations with each time step in 6 femtosecond. We recorded the atomic configuration, and stress conditions were recorded at each tensile loading for further analysis.

## 3. Results and Analysis

In this section, we present atomistic configurations, stress fields and stress-strain curves of LEC and REC models for clarifying the microstructure evolution and the effect of the TB spacing and crack position on crack propagation. The relationship between dislocation density and fracture toughness is also investigated.

### 3.1. Left Edge Crack (LEC) Model vs. Right Edge Crack (REC) Model

**Figure 2:**
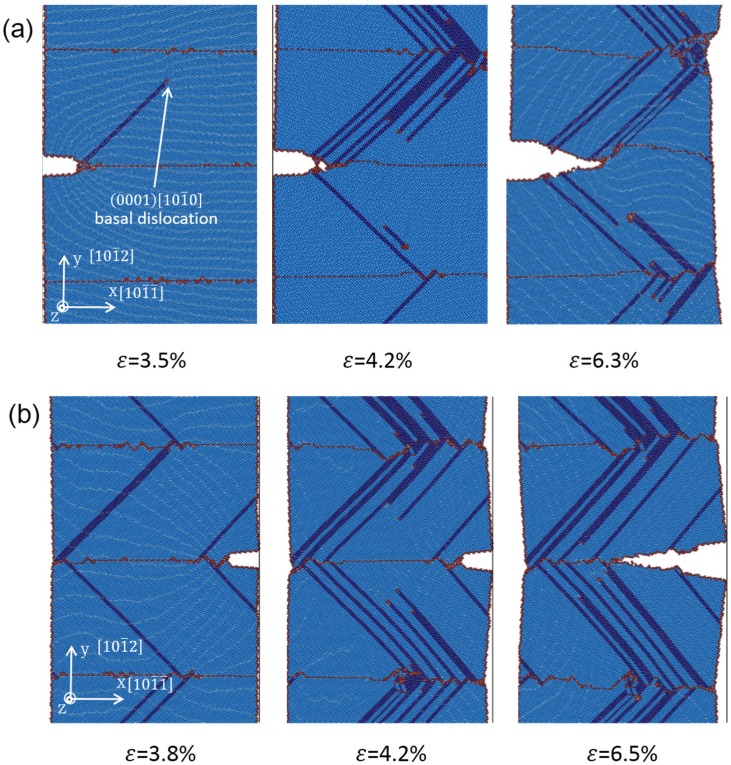
The atomistic configurations of (**a**) LEC model and (**b**) REC model at 5 K with the TB spacing of 15.5 nm.

Figure 2 shows the atomistic configurations of LEC and REC models with TB spacing of 15.5 nm under various tensile strains. In the LEC model (Figure 2a), the first (0001) $\left[10\bar{1}0\right]$ partial dislocation is observed to emit at the junction of crack tip and TB interface under 3.5% tensile strain. Subsequently, partial dislocation emissions are observed from the neighboring TB interfaces. With the strain increasing, dislocation emissions both at the crack tip and the TBs are observed accompanying the LEC propagations. Voids induced by the dislocation motions are captured ahead of the crack tip under 4.2% tensile strain. In the REC model (Figure 2b), it is found that the crack tip is sharper than that in the LEC model after crack propagation. Moreover, we can only observe dislocation emission at the TB interface, but no dislocation emission at the crack tip. With the strain increasing, most of the dislocations ahead of the crack are originated from the neighboring TBs during the propagation process. Besides, a few dislocations generated at the crack tip induce slight coherency loss of TBs.

In order to investigate the different crack propagation and dislocation emission mechanisms for LEC and REC models, the energy release rate for dislocation nucleation at the crack tip is calculated. Considering that basal dislocation is the only dislocation activated at the beginning of the deformation, we calculate the energy release rate for the basal dislocation nucleation based on the Rice model [28]. The formula is given as follow:

(1) $$G_{disl}=8\gamma_{usf}[1+(1-\nu )\tan^{2}\varphi ]/[(1+\cos\theta )\sin^{2}\theta ]$$

where γ*_usf_* is the unstable stacking fault energy; ν is Poisson’s ratio of magnesium; φ is the angle between Burgers vector and a line in the slip plane perpendicular to the crack front; θ is the angle between the slip plane and the crack surface. It is worth noting the aforementioned formula is based on the perfect sharp crack model. Though the LEC and REC employed in our simulations are not atomically sharp at the beginning of the simulation, they both possess a sharp crack tip after propagation. Thus it is suitable to roughly compare dislocation motions at the LEC tip with those at the REC tip using this formula. Table 2 lists the energy cost for dislocation generation along the basal plane.

**Table 2:** Energy cost for dislocation generation along the basal plane. The value of parameters γ*_usf_* is taken from the work of Muzyk *et al.* [29]. The Poisson’s ratio is captured from typical experimental values, and the rest of the parameters are calculated based on the same interatomic potential used in present work.

Crack	*ν*	*ɸ*	*θ*	γ *_usf_* (*J*/*m*^2^)	*G_disl_* (*J*/*m*^2^)
LEC	0.31	0	43	0.092	0.914
REC	0.31	0	137	0.092	5.888

Comparing the energy release rate for basal dislocation nucleation in the LEC and REC models, it is obvious that the partial dislocations are favorable to emit from the crack tip in the LEC model. This result is consistent with the phenomena observed in our simulation that LEC propagates accompanying the dislocation emission at the crack tip (Figure 2a). LEC propagation can be described in four steps: (i) the basal dislocation emits at the crack tip; (ii) voids are generated ahead of the crack tip due to the dislocation motion; (iii) voids coalescet with the crack which results in the crack propagation initiation; (iv) atomic decohesion occurs which sharpens the crack tip. This process indicates that the crack propagation in the LEC model experiences a ductile to brittle transition from dislocation emission at the crack tip to crack surface cleavage due to atomic decohesion.

**Figure 3:**
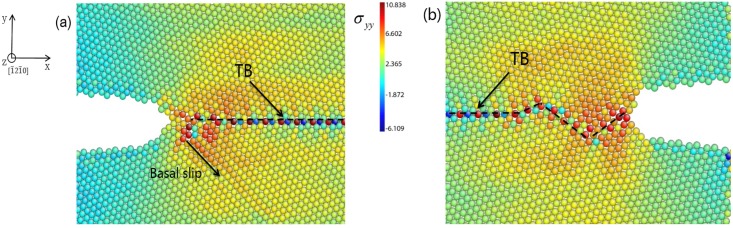
Tensile stress δ*_yy_* distribution around crack tips in (**a**) LEC model under 4% strain and (**b**) REC model under 6.2% strain respectively at onset of crack propagation.

The situation in the REC model is not exactly same. During the crack growth process, dislocation emissions from the crack tip are seldom observed (Figure 2b). The crack propagation can be only attributed to the atomic decohesion. The crack tip is directly sharpened at the beginning of the crack growth. Further evidence is accessed by plotting tensile stress distribution around the crack tip in the LEC and REC models respectively at the onset of the crack growth in Figure 3. The stress concentration is located at about 3 nm away from the crack tip in the LEC model (Figure 3a). Moreover, the direction of stress concentration is along the basal slip plane. It can be expected that the basal dislocation emission is preferred over the crack cleavage in the LEC model. In the REC model, the stress concentration is very close to the crack tip along the incoherent TB (Figure 3b). Thus the high tensile stress can directly induce a new free surface nucleation at the crack tip along the TB interface, which induces the brittle crack propagation (Figure 2b). Therefore, the mechanisms of crack growth in LEC and REC models are not exactly the same due to the different microstructure evolutions at the crack tip.

### 3.2. Effect of TB Spacing on Crack Propagation

#### 3.2.1. Stress-Strain Curves

**Figure 4:**
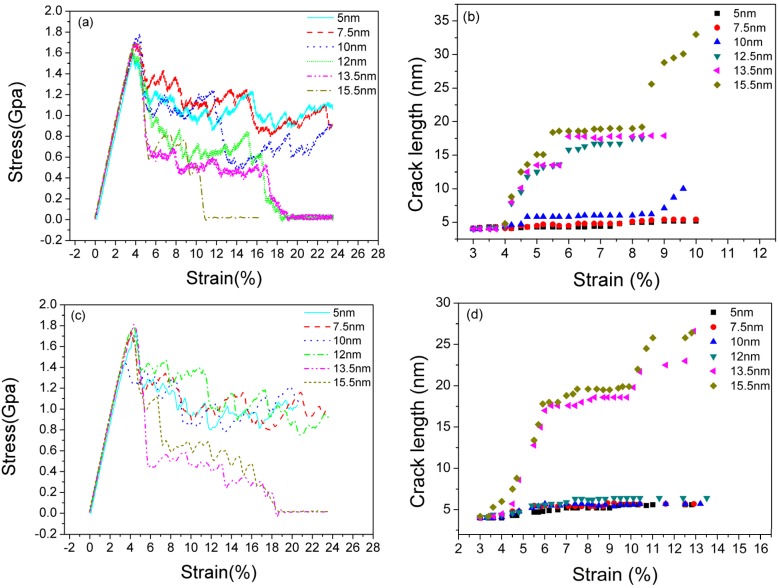
The stress-strain curves with different TB spacings and the variation of crack length with increasing strain. (**a,b**): LEC models; (**c,d**): REC models.

Figure 4a shows the typical stress-strain curves of LEC models with different TB spacings ranging from 5 to 15.5 nm. The variation of the stress-strain relationship induced by different TB spacings is remarkable. When the TB spacing is below 10 nm, the flow stress is clearly higher than that of samples with a TB spacing above 10 nm. Moreover, when the TB spacing rises beyond 10 nm, significant stress droppings are observed in LEC models. We plot the curve of the crack length *vs.* strain for five TB spacing samples in Figure 4b. Crack propagation is effectively impeded in 5 nm and 7.5 nm spacing samples. Remarkable crack propagation can be observed until the spacing is over 10 nm, when it corresponds to a stress dropping on the stress-strain curves. The similar situation happens in REC models. The sharp stress droppings can be only observed in 13.5 nm and 15.5 nm spacing models (Figure 4c). Crack propagations also happen at the onset of stress dropping in these two models (Figure 4d). When the TB spacing is smaller than 13.5 nm in REC models, the flow stress presents a relatively stable state and no obvious crack growth is observed. Reviewing all the samples in LEC and REC models, brittle to ductile transition is observed by decreasing the TB spacing. We propose there is a critical value for this transition, which is 10 nm in LEC models and 13.5 nm in REC models, respectively. When TB spacing is below the critical value, samples represent ductile properties where the crack growth is impeded by dislocation emissions.

#### 3.2.2. Atomistic Configurations

**Figure 5:**
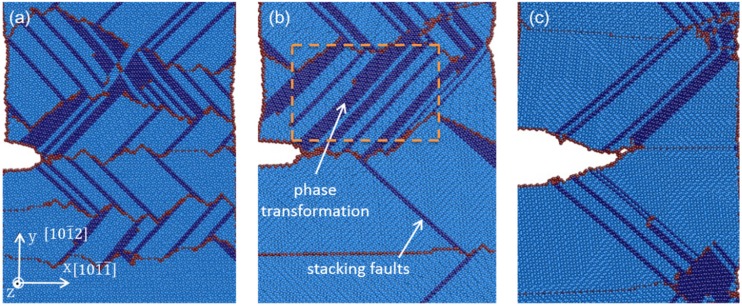
The atomistic configurations of LEC model under 6% tensile strain at 5 K with TB spacing (**a**) 5 nm; (**b**) 10 nm and (**c**) 15.5 nm.

Figure 5a–c shows atomistic configuration of crack tips under 6% strains with different TB spacing (5 nm, 10 nm and 15.5 nm) in LEC models. In all three samples, stacking faults along $\left[\bar{1}010\right]$ and $\left[10\bar{1}0\right]$ directions are observed, accompanying dislocation emission from the TBs and crack tip. Due to the coalescence of basal plane stacking faults, phase transformations from original HCP structure to FCC structure are found. Stacking faults and phase transformation are the main plastic deformation mechanisms in all six LEC samples.

**Figure 6:**
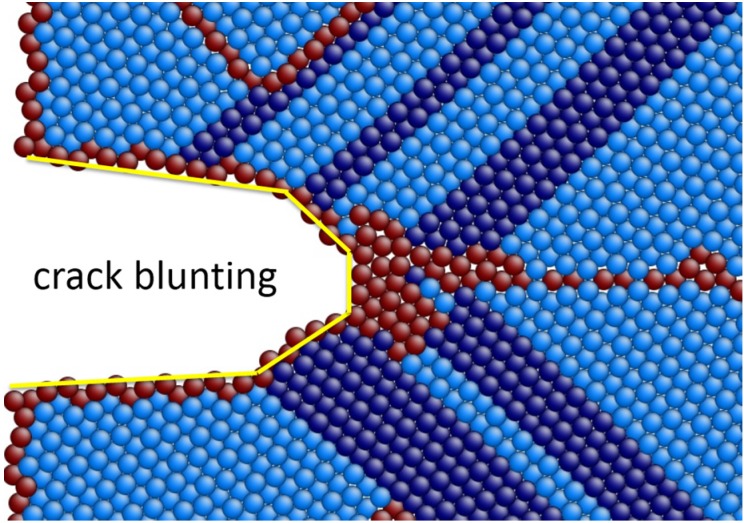
Details of the atomistic configuration around the crack tip under 8% tensile strain with 5 nm TB spacing in the LEC model, showing that the crack tip is significantly blunted because of the dislocation emission and motion.

Moreover, the region of stacking faults and phase transformation in Figure 5a,b is significantly larger than that in Figure 5c. When the TB spacing is below 10 nm (including 10 nm sample), more stacking faults and phase transformations are formed due to drastic dislocation motions around the crack tip. Instead of generating voids ahead of the crack, dislocations piling up at the crack tip and emissions from the crack ledge significantly blunt the crack tip, which results in the enlarging of the crack width rather than length (Figure 6). As a result, crack propagations are impeded. This phenomenon is consistent with the description of the stress-strain curves in Figure 4a whereby no considerable stress dropping in 5 nm and 7.5 nm samples is observed. Meanwhile, TB migrations associated with the interaction between dislocations and TBs are more intense. Thus stacking faults, phase transformation and TB migration dominate the plastic deformation when TB spacing is below 10 nm. According to Zhu *et al.* [30], the coherency loss of the TBs also attributes to enhanced ductility, it is assumed that NT magnesium samples present ductile properties with a TB spacing below 10 nm.

When the TB spacing is over 10 nm, crack cleavage is observed (Figure 5c). This means there is a competition between crack propagation and plastic deformation at the crack tip with the variation of the TB spacing. When the TB spacing is over 10 nm, crack cleavage takes priority over stacking faults emission and phase transformation. As a result, fracture toughness is weakened by the crack propagations.

**Figure 7:**
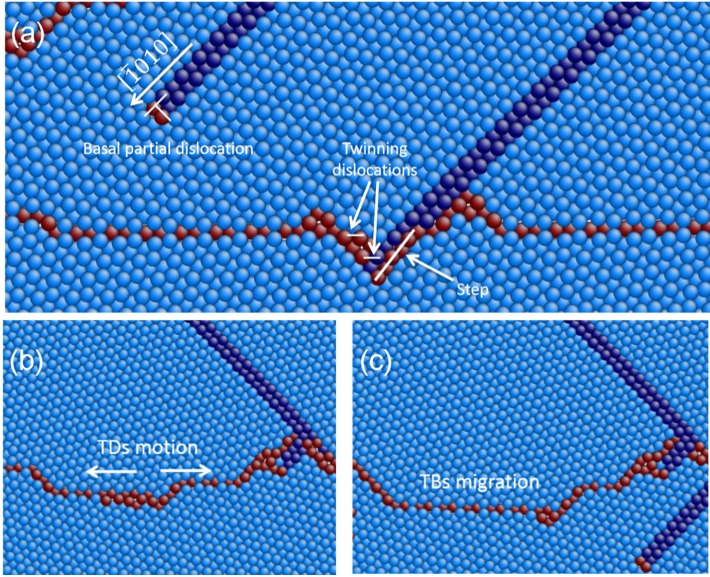
(**a**) A basal partial dislocation emits from TB followed by stacking faults, and the basal dislocation and stacking fault are arrested by TB; (**b**) TDs gliding along the TB interface; and (**c**) TB migration.

In Figure 5, TB motion and migration are also clearly observed due to the formation of stacking faults and phase transformation. In Figure 7a, the detail of a basal partial dislocation followed by a stacking fault emitting from TB is presented. Moreover, the partial dislocation and the stacking fault are arrested by the TB, leaving a step and twinning dislocations (TDs) at the TB interface, which is also in agreement with the description by Serra *et al.* [31]. In Figure 7b,c, the detail of TB migration is presented, which is due to TDs gliding along the TB interface.

**Figure 8:**
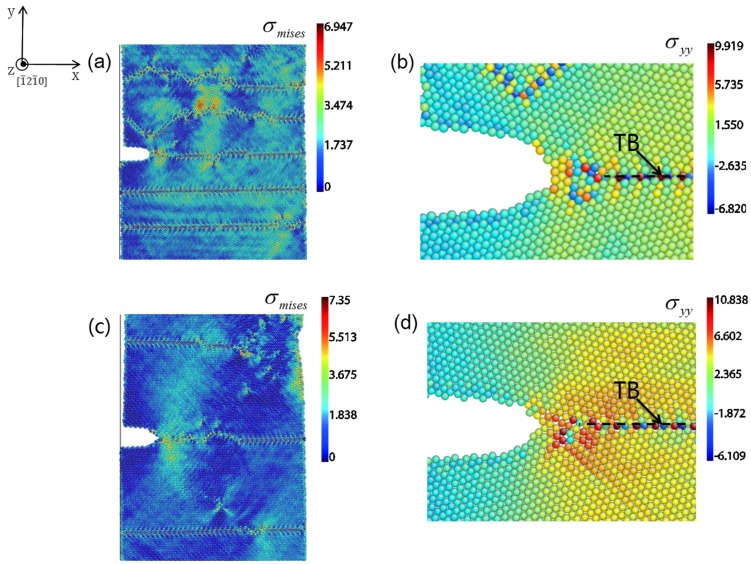
Stress fields of 5 nm and 15.5 nm samples around crack tips under 6% strain in LEC model respectively. (**a**) Von Mises stress δ*_mises_* distribution; and (**b**) tensile stress δ*_yy_* distribution in 5 nm sample; (**c**) Von Mises stress δ*_mises_* distribution; and (**d**) tensile stress δ*_yy_* distribution in 15.5 nm sample.

To reveal the relationship between local stress concentration and plastic deformation at crack tip, we plot the distribution of von Mises stress δ*_mises_* and tensile stress δ*_yy_* in Figure 8a–d for 5 nm and 15.5 nm samples under 6.2% strain in LEC model, respectively. The von Mises stress is for isotropic materials, but a rough estimate for the location of plastic deformation in our present work is still reasonable. The von Mises stress δ*_mises_* has three peak value points along the TB interfaces in the 5 nm sample (Figure 8a). One of the stress concentration sites is located 1.5 nm ahead of the crack tip along the TBs and the other two lie on the neighboring TB interface, due to the piling up of dislocations in TBs. Figure 8b shows the tensile stress δ*_yy_* distribution around the crack tip in the 5 nm spacing sample. The peak value is about 9 Gpa and 4.5 nm away from the crack tip. However, the stress concentration in this region is not as intense since the sufficient dislocation motions can effectively release it. Thus a new surface is hardly created by the weak stress concentration ahead of the crack tip. Instead, crack blunting occurs by the dislocation emission. In contrast to the 5 nm spacing sample, the situation in the 15.5 nm spacing sample is different. The peak value of δ*_mises_* is mainly located at the junction of the crack and TB (Figure 8c). Less dislocation motions in large spacing samples result in the difficulty of releasing stress concentration around the crack tip. Figure 8d shows the corresponding tensile stress δ*_yy_* distribution which has the peak value as high as 10.8 Gpa and 3 nm away from the crack tip. The stress concentration ahead of the crack tip is much more intense, which provides chances for voids generating at this location. Therefore, crack propagation occurs by the coalescence of voids with the crack. TBs favor serving as a cleavage plane in the large TB spacing samples, which is consistent with other studies [32,33]. Thus, the large spacing sample exhibits brittle properties for the crack propagation.

**Figure 9:**
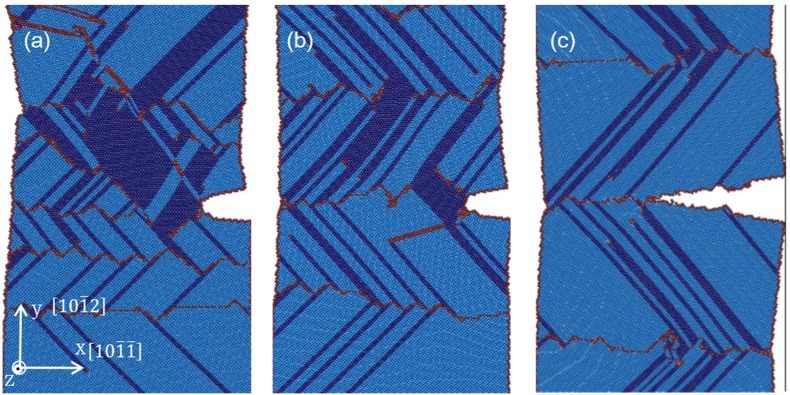
The atomistic configurations of REC models under 6.5% tensile strain at 5 K, the TB spacing (**a**) 5 nm; (**b**) 12 nm; (**c**) 15.5 nm.

Figure 9a–c shows atomistic configurations of crack tip under various strains with different TB spacings (5 nm, 12 nm and 15.5 nm) in REC models. When the TB spacing is below 13.5 nm (Figure 9a,b), crack growth is impeded due to phase transformation at the crack tip which is induced by dislocation emissions from the neighboring TBs. Moreover, the TB interface ahead of the crack which originally serves as the crack plane is almost annihilated due to the dislocation motions and phase transformations, and the crack tip is blunted. When the TB spacing is above 13.5 nm (Figure 9c), less dislocations are observed, and no remarkable phase transformation ahead of the crack tip are found. Thus brittle cleavage is captured which badly deteriorates the fracture toughness of these samples. However, with the strain increasing, weak phase transformation can still be observed accompanying crack growth, which prevents the excessively quick propagation of the crack. Compared with LEC models, the critical TB spacing for ductile to brittle transition is larger in REC models.

### 3.3. Dislocation Density vs. Fracture Toughness

**Figure 10:**
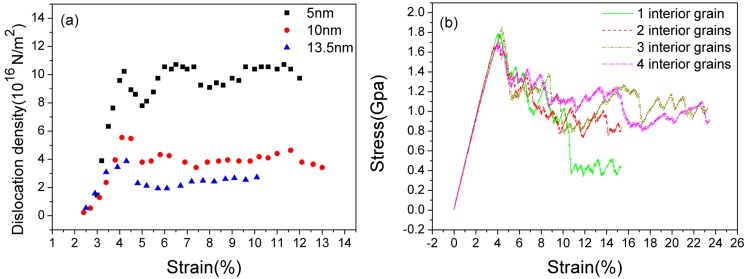
(**a**) The trend of dislocation density with strain increasing by choosing three typical TB spacings in the LEC model; (**b**) The stress-strain curves of LEC models with different interior grains with a constant TB spacing.

In LEC and REC models, the dislocation motion originating from TBs plays a crucial role in crack propagation behaviors. As a matter of fact, different TB spacings stands for different dislocation densities since TBs serve as the main dislocation generation source. By calculating the number of the dislocation cores per square meter, we plot the trend of dislocation density with strain increasing by choosing three typical TB spacings (5 nm, 10 nm and 13.5 nm) in the LEC model. In Figure 10a, the dislocation density in the 5 nm sample ranges from 0.8 × 10^17^ to 1.1 × 10^17^ m^−2^. It is much higher than those in 10 nm and 13.5 nm samples which are approximately 0.40 × 10^17^ m^−2^ and 0.25 × 10^17^ m^−2^, respectively. Dislocation density *vs.* strain scatter diagram illustrates that a smaller TB spacing induces a higher dislocation density. Comparing with the trend of crack length *vs.* strain in Figure 5b, we conclude that a low dislocation density condition may favor the crack propagation of NT magnesium.

To further verify the conclusion above, we built type B models with different numbers of interior grains but a constant TB spacing of 7.5 nm. The crack employed in this model is LEC. According to simulation results in Section 3.1, no remarkable stress dropping is observed on the stress-strain curve of an LEC sample in type A with 7.5 nm TB spacing below a tensile strain of 20%, which means pre-existing crack propagation is impeded. When we reduce the number of interior grains, the situation seems to be changed. We only plot the stress-strain curves for 1–4 interior grain samples to demonstrate the competition between dislocation motion and crack growth. In Figure 10b, average flow stress decreases with the reduction of the grain numbers. There is a remarkable stress dropping under 10% strain in the one-grain sample, which indicates the crack propagation is obtained. The reason for this stress dropping on a 7.5 nm spacing sample is mainly due to the lack of dislocation nucleation and motion. The stress concentration at the crack tip is not effectively released by stacking dislocation emission, thus the crack initiates propagation with the strain increasing in the one-grain sample. Therefore, a higher dislocation density, namely more TBs and smaller TB spacing, can enhance the fracture toughness of NT magnesium.

## 4. Conclusions

In the present work, we performed a series of MD simulations to investigate the mechanical behaviors and plastic deformation mechanisms of crack in $\{10\bar{1}2\}<10\bar{1}\bar{1}>$ NT magnesium. TB spacing variations are considered as revealing their effects on the fracture toughness. Main conclusions are listed as follows:

(1) Basal dislocation nucleates and emits from TBs and crack tip in $\{10\bar{1}2\}<10\bar{1}\bar{1}>$ NT magnesium. Stacking faults and phase transformation are main deformation mechanisms. Moreover, TBs effectively hinder the dislocation motion and reserve dislocations to accommodate plastic strain. Because of the TDs gliding along the TB interface, TB migration is obtained.

(2) Different crack propagation behaviors are observed for samples with different crack positions (LEC and REC). By calculating the energy release rate for dislocation nucleation at the crack tip, it is indicated that dislocation motions more favorably occur in the LEC tip. Thus in the LEC model, the formation of voids which result from the dislocation emission ahead of a crack tip initiates a ductile fracture. The LEC growth experiences a transition from a ductile to brittle pattern, whereas for the REC model, a brittle cleavage is observed due to the suppression of dislocation emission at the crack tip.

(3) The ductile to brittle transition is found to mainly depend on the competition between dislocation motion and crack growth. TB spacing plays an important role in this competition mechanism. In LEC models when the spacing is below 10 nm, dislocation emission, stacking faults and phase transformation dominate the plastic deformation, thus the crack tip is blunted due to the dislocation motions. When the spacing is above 10 nm, crack cleavage is more likely. In REC models, the critical TB spacing is about 13.5 nm, which is larger than that in LEC models.

(4) It is revealed that the dislocation density changed by the TB spacing variation accounts for the ductile to brittle transition. Decreasing TB spacing leads to the rise of dislocation densities, which provides more dislocation motions to accommodate the plastic deformation and suppress the crack growth. Thus a better fracture toughness of $\{10\bar{1}2\}<10\bar{1}\bar{1}>$ NT magnesium can be realized by enhancing the dislocation densities.

## References

[1] Dalla Torre F., Lapovok R., Sandlin J., Thomson P.F., Davies C.H.J., Pereloma E.V. (2004). Microstructures and properties of copper processed by equal channel angular extrusion for 1–16 passes. Acta Mater..

[2] Iwasaki H., Higashi K., Nieh T.G. (2004). Tensile deformation and microstructure of a nanocrystalline Ni-W alloy produced by electrodeposition. Scr. Mater..

[3] Meyers M.A., Mishra A., Benson D.J. (2006). Mechanical properties of nanocrystalline materials. Prog. Mater. Sci..

[4] Wang Y.M., Sansoz F., LaGrange T., Ott R.T., Marian J., Barbee T.W., Hamza A.V. (2013). Defective twin boundaries in nanotwinned metals. Nat. Mater..

[5] Mirshams R.A., Xiao C.H., Whang S.H., Yin W.M. (2001). R-curve characterization of the fracture toughness of nanocrystalline nickel thin sheets. Mater. Sci. Eng. A.

[6] Li Y.S., Tao N.R., Lu K. (2008). Microstructural evolution and nanostructure formation in copper during dynamic plastic deformation at cryogenic temperatures. Acta Mater..

[7] Zhang X., Wang H., Chen X.H., Lu L., Lu K., Hoagland R.G., Misra A. (2006). High-strength sputter-deposited cu foils with preferred orientation of nanoscale growth twins. Appl. Phys. Lett..

[8] Sun H.Q., Shi Y.N., Zhang M.X., Lu K. (2007). Plastic strain-induced grain refinement in the nanometer scale in a Mg alloy. Acta Mater..

[9] Yu Q., Qi L., Chen K., Mishra R.K., Li J., Minor A.M. (2012). The nanostructured origin of deformation twinning. Nano Lett..

[10] Pozuelo M., Mathaudhu S.N., Kim S., Li B., Kao W.H., Yang J.M. (2013). Nanotwins in nanocrystalline Mg-Al alloys: An insight from high-resolution tem and molecular dynamics simulation. Philos. Mag. Lett..

[11] Lu L., Chen X., Huang X., Lu K. (2009). Revealing the maximum strength in nanotwinned copper. Science.

[12] Song B., Guo N., Liu T.T., Yang Q.S. (2014). Improvement of formability and mechanical properties of magnesium alloys via pre-twinning: A review. Mater. Des..

[13] Song H.Y., Li Y.L. (2012). Effect of twin boundary spacing on deformation behavior of nanotwinned magnesium. Phys. Lett. A.

[14] Qin E.W., Lu L., Tao N.R., Lu K. (2009). Enhanced fracture toughness of bulk nanocrystalline Cu with embedded nanoscale twins. Scr. Mater..

[15] Hong S.G., Park S.H., Lee C.S. (2010). Role of {10–12} twinning characteristics in the deformation behavior of a polycrystalline magnesium alloy. Acta Mater..

[16] Song B., Xin R.L., Sun L.Y., Chen G., Liu Q. (2013). Enhancing the strength of rolled ZK60 alloys via the combined use of twinning deformation and aging treatment. Mater. Sci. Eng. A.

[17] Tang T., Kim S., Horstemeyer M.F. (2010). Fatigue crack growth in magnesium single crystals under cyclic loading: Molecular dynamics simulation. Comput. Mater. Sci..

[18] Serra A., Bacon D.J., Pond R.C. (2002). Twins as barriers to basal slip in hexagonal-close-packed metals. Metall. Mater. Trans. A.

[19] Christian J.W., Mahajan S. (1995). Deformation twinning. Prog. Mater. Sci..

[20] Wu X.L., Qi Y., Zhu Y.T. (2007). Partial-mediated slips in nanocrystalline Ni at high strain rate. Appl. Phys. Lett..

[21] Liu X.Y., Adams J.B., Ercolessi F., Moriarty J.A. (1996). Eam potential for magnesium from quantum mechanical forces. Model. Simul. Mater. Sci. Eng..

[22] Kim D.H., Ebrahimi F., Manuel M.V., Tulenko J.S., Phillpot S.R. (2011). Grain-boundary activated pyramidal dislocations in nano-textured Mg by molecular dynamics simulation. Mater. Sci. Eng. A.

[23] Kim D.H., Manuel M.V., Ebrahimi F., Tulenko J.S., Phillpot S.R. (2010). Deformation processes in-textured nanocrystalline Mg by molecular dynamics simulation. Acta Mater..

[24] Li B., Ma E. (2009). Zonal dislocations mediating twinning in magnesium. Acta Mater..

[25] Plimpton S. (1995). Fast parallel algorithms for short-range molecular dynamics. J. Comput. Phys..

[26] Faken D., Jónsson H. (1994). Systematic analysis of local atomic structure combined with 3D computer graphics. Comput. Mater. Sci..

[27] Li J. (2003). Atomeye: An efficient atomistic configuration viewer. Model. Simul. Mater. Sci. Eng..

[28] Rice J.R. (1992). Dislocation nucleation from a crack tip: An analysis based on the peierls concept. J. Mech. Phys. Solids.

[29] Muzyk M., Pakiela Z., Kurzydlowski K.J. (2012). Generalized stacking fault energy in magnesium alloys: Density functional theory calculations. Scr. Mater..

[30] Zhu T., Li J., Samanta A., Kim H.G., Suresh S. (2007). Interfacial plasticity governs strain rate sensitivity and ductility in nanostructured metals. Proc. Natl. Acad. Sci..

[31] Serra A., Bacon D.J. (1996). A new model for {1012} twin growth in hcp metals. Philos. Mag. A.

[32] Jang D., Li X., Gao H., Greer J.R. (2012). Deformation mechanisms in nanotwinned metal nanopillars. Nat. Nano.

[33] Yuan F.P., Wu X.L. (2013). Atomistic scale fracture behaviours in hierarchically nanotwinned metals. Philos. Mag..

